# A different vision of translational research in biomarker discovery: a pilot study on circulatory mitochondrial proteins as Parkinson’s disease potential biomarkers

**DOI:** 10.1186/s40035-020-00188-0

**Published:** 2020-04-03

**Authors:** Sandra I. Anjo, Patrícia Valério dos Santos, Luiza Rosado, Graça Baltazar, Inês Baldeiras, Diana Pires, Andreia Gomes, Cristina Januário, Miguel Castelo-Branco, Mário Grãos, Bruno Manadas

**Affiliations:** 1 CNC - Center for Neuroscience and Cell Biology, University of Coimbra, Coimbra, Portugal; 2 Faculty of Medicine, University of Coimbra, Coimbra, Portugal; 3 Centro de Investigação em Ciências da Saúde (CICS-UBI), Universidade da Beira Interior, Covilhã, Portugal; 4 Centro Hospitalar Cova da Beira, E.P.E, Covilhã, Portugal; 5 Neurology Department, Centro Hospitalar e Universitário de Coimbra, Coimbra, Portugal; 6 Coimbra Institute for Biomedical Imaging and Translational Research (CIBIT), Coimbra, Portugal; 7 Biocant, Biotechnology Transfer Association, Cantanhede, Portugal; 8 Institute for Interdisciplinary Research, University of Coimbra (IIIUC), Coimbra, Portugal

**Keywords:** Mitochondrial-related proteins, SWATH-MS, Parkinson’s disease, Biomarker discovery, Blood-biomarker, Secretomes, Oxidative stress

## Abstract

**Background:**

The identification of circulating biomarkers that closely correlate with Parkinson’s Disease (PD) has failed several times in the past. Nevertheless, in this pilot study, a translational approach was conducted, allowing the evaluation of the plasma levels of two mitochondrial-related proteins, whose combination leads to a robust model with potential diagnostic value to discriminate the PD patients from matched controls.

**Methods:**

The proposed translational approach was initiated by the analysis of secretomes from cells cultured under control or well-defined oxidative stress conditions, followed by the identification of proteins related to PD pathologic mechanisms that were altered between the two states. This pipeline was further translated into the analysis of undepleted plasma samples from 28 control and 31 PD patients.

**Results:**

From the secretome analysis, several mitochondria-related proteins were found to be differentially released between control and stress conditions and to be able to distinguish the two secretomes. Similarly, two mitochondrial-related proteins were found to be significantly changed in a PD cohort compared to matched controls. Moreover, a linear discriminant model with potential diagnostic value to discriminate PD patients was obtained using the combination of these two proteins. Both proteins are associated with apoptotic mitochondrial changes, which may correspond to potential indicators of cell death. Moreover, one of these proteins, the VPS35 protein, was reported in plasma for the first time, and its quantification was only possible due to its previous identification in the secretome analysis.

**Conclusions:**

In this work, an adaptation of a translational pipeline for biomarker selection was presented and transposed to neurological diseases, in the present case Parkinson’s Disease. The novelty and success of this pilot study may arise from the combination of: i) a translational research pipeline, where plasma samples are interrogated using knowledge previously obtained from the evaluation of cells’ secretome under oxidative stress; ii) the combined used of statistical analysis and an informed selection of candidates based on their link with relevant disease mechanisms, and iii) the use of SWATH-MS, an untargeted MS method that allows a complete record of the analyzed samples and a targeted data extraction of the quantitative values of proteins previously identified.

## Background

A conventional pipeline for biomarker discovery usually contemplates the collection of clinical samples, such as blood, and their analysis by mass spectrometry. Distinct statistical methods are then applied to identify the candidates that better differentiate the groups being studied, the so-called potential biomarkers. Finally, the candidates highlighted are validated in a different cohort [1]. However, clinical samples, in particular the case of plasma and serum (the primary source for biomarker discovery), are known to have a large dynamic range, which makes it difficult to reach the less abundant species. Thus, to avoid missing out some critical candidates, a highly demanding sample processing is required to reduce the complexity of the samples. Also, large cohorts are needed to perform biomarker discovery directly from human samples in order to overcome the large biological variability intrinsic of these samples [1–4]. All these aspects combined result in a very challenging analysis, which may justify, in part, the low reproducibility and success of biomarker discovery studies. In line with this general tendency, the studies devoted to the identification of circulating biomarkers for Parkinson’s Disease (PD) have also failed several times in the past [5, 6].

In the present work, instead of performing the discovery process directly from the clinical samples, the biomarker selection was first tested in cells’ secretomes collected under well-defined and controlled conditions. Secreted proteins are key players in cellular communication and the regulation of many physiological processes; therefore, being good predictors of the cellular state and an important source of biomarkers [7, 8]. In fact, translational screenings have been successfully applied in other areas, in particular, cancer and cardiovascular research [9]. An additional step to this conventional translational pipeline was also introduced by the combination of statistical analysis with an informed selection of the biomarker candidates based on relevant biological functions (as it was recently proposed [10]).

Moreover, the improvements in mass spectrometry acquisition methods, in particular the introduction of the SWATH-MS, a data-independent acquisition method capable of extracting quantitative information from large data sets, could be extremely beneficial for this type of translational studies. Therefore, SWATH-MS was used in this work to take advantage of: i) its capacity to obtain a complete record of each sample (resulting in a “digital biobank”) which can be reanalyzed indefinitely, and ii) to get a spectra library that can be used in different sample sets. Thus, it is an important tool for prospective and integrative studies [11].

Therefore, this pilot study aimed todemonstrate the potential of translational pipelines for the identification of circulating biomarker candidates of neurodegenerative diseases such as PD.

## Methods

### Characterization of the cellular model used to study the impact of oxidative stress in cells’ secretome

The experimental conditions used to obtain the HeLa cells’ secretomes were selected based on the profile of activation of two survival pathways modulated by oxidative stress, ERK1/2, and Akt pathways. Their activation was studied as described by Ruffels et al. [12], where the activation of distinct kinases in SH-SY5Y cells was induced by hydrogen peroxide (H_2_O_2_). Pathways activation was verified by immunoblot against the phosphorylated and total forms of ERK1/2 and Akt, and the protein GAPDH was used as a loading control (see Supplementary Methods – Table 1 for detailed information). Additionally, different parameters were also assessed to characterize the impact of oxidative stress caused by the selected condition (40 min stimulation with 1 mM H_2_O_2_). Cell Titer Glo® Luminescent Cell Viability Assay (Promega) was used to measure the metabolically active cells by quantitation of the cellular ATP levels. Membrane integrity (cytolysis) and cytotoxicity were evaluated using a Lactate dehydrogenase (LDH) cytotoxicity assay kit (Pierce, Thermo Scientific) to measure LDH released from damaged cells and the CellTox™ Green Cytotoxicity Assay (Promega) which stains the dead cells’ DNA, respectively. Finally, oxidative stress detection was performed by measuring the reactive oxygen species (ROS) using CellROX® Orange Reagent (Invitrogen). ATP and ROS levels were assessed immediately after stimulation and 24 h after stimulation (the condition used to obtain the secretome). For the 24 h measurement, the medium was changed to a new DMEM medium without FBS after the stimulation, and cells were allowed to recover for 24 h. Each condition was performed in a total of four replicates.

Statistical analyses were performed using IBM® SPSS® Statistics Version 22. Single comparisons between the different conditions studied were made using the Wilcoxon Signed-Rank test. The level of significance in all the statistical analyses was set at *p* < 0.05.

### Secretome analysis

HeLa cells secretomes were obtained as described in [13]. Briefly, cells were seeded at 12⨯10^3^ cells/cm^2^ in four 55 cm^2^ plates, and 48 h later the culture medium [DMEM supplemented with 10% (v/v) FBS] was discarded and cells were washed twice with warm PBS to remove the remaining FBS. Then, the culture medium was changed to DMEM without FBS (control condition) or 1 mM of H_2_O_2_ in DMEM without FBS (the stress condition) for 40 min, after which the medium was changed again to DMEM without FBS. The conditioned media were collected 24 h after the stimulation, and the same amount (2 μg) of a recombinant protein (MBP-GFP) was added to each media immediately after collection, to be used as the internal standard (IS) in the quantitative analysis. Each condition was performed in a total of four replicates. The collected secretomes were concentrated, followed by protein precipitation of the samples. The protein pellets were dissolved in Laemmli buffer, and the entire samples were subjected to in-gel digestion using the Short-GeLC [14] for subsequent quantitative analysis by SWATH-MS [11]. Additionally, one plate of each biological replicate was combined to create a pooled sample for each secretome. These pooled samples were spiked with the recombinant protein and digested using the same condition of the individual replicates and were utilized for information-dependent acquisition (IDA) experiments to build a specific protein library to be used in SWATH-MS analysis. Secretome samples were analyzed on an AB Sciex 5600 TripleTOF in two modes: IDA for protein identification and library generation, and SWATH acquisition (see SWATH windows at Supplementary Methods – Table 2) for quantitative analysis as previously described [13] and detailed in the Supplementary Methods. False Discovery Rate (FDR) analyses were used to assess the quality of protein identification (for library generation) and protein quantification. Positive identifications were considered when identified proteins and peptides reached a 5% local FDR [15, 16], and the SWATH quantitation was attempted for all proteins in library files that were identified below 5% local FDR (i.e., 95% confidence). Protein levels were estimated based on peptides that met the 1% FDR threshold in at least three biological replicates, and the peak areas of the target fragment ions of those peptides were extracted across the experiment using a 4 min extracted-ion chromatogram (XIC). Protein levels were estimated by summing all the transitions from all the peptides for a given protein (an adaptation of [17]) and further normalized to the levels of the IS (the protein MBP-GFP).

One-sample Student’s *t*-test against a theoretical value of one was applied to the protein ratios in the secretome analysis. The level of significance in all the statistical analyses was set at *p* < 0.05. Statistical analyses were performed using IBM® SPSS® Statistics Version 22.

### Proteomics analysis of plasma samples

To evaluate the feasibility of the proposed pipeline, the results obtained from the secretome assay were further translated to the analysis of human samples (Table 1 and Supplementary Table 1 ). This part of the study was carried out using samples from 31 PD patients, 17 males (54.8%) and 14 females (45.2%), aged between 65 and 86 years. PD patients were recruited from the appointment list of Centro Hospitalar Cova da Beira (CHCB, Covilhã - Portugal) Neurology sector, between January 2013 and December 2013. Patients with a clinical diagnosis of PD established by the UKPDBBC and modified Hoehn & Yahr staging scale between 1 and 4 were included. All patients underwent an interview with a questionnaire, neurological examination performed by a neurologist, laboratory tests, and medical record evaluation. Participants with Diabetes mellitus, hypertension, coronary and heart disease, hyperlipidemia, and thyroid disease were included, if treated with the appropriate medication and if there was no evidence of exacerbations or complications in the clinical records, in the past 6 months. The control group had 28 participants, 16 males (57.1%) and 12 females (42.9%), aged between 55 and 83 years. They were all volunteers from CHCB. Most of them were recruited from the Urology and Gynecology sectors. All volunteers underwent an interview with a questionnaire, neurological and physical exam, laboratory tests, and medical records evaluation. A summary neurological examination was performed to exclude neurological symptoms, and cardiac and pulmonary assessement provided additional clinical information. Interview and medical record evaluation allowed to obtain comorbidities and actual medications. All documents were identified by a study number to maintain confidentiality. The Ethical Committee of CHCB approved the research protocol. Patients and controls gave their informed consent to the study.

**Table 1:** Groups’ demographic characterization and overview of the clinical data of the Parkinson’s disease group used for the discovery study

	Age at blood coll. (y)^a^	Gender^b^	Disease Duration (y)	Age at Onset (y)	Family history^c^	HY scale^d^	Total L-Dopa (converted CR + IR) (mg/day)^e^
**Parkinson's Disease Group**	74 ± 7 [63, 83]	M:17 (54.8%); F: 14 (45.2%)	6.2 ± 3.8 [0.7, 15]	67.7 ± 7.4 [48, 80]	N: 30; Y:1	1.8 ± 1 [1, 4]	374.7 ± 288.7 [0, 1150]
**Control Group**	67 ± 10 [55, 83]	M: 16 (57.1%) F: 12 (42.9%)	–	–	–	–	–

^a^Age at blood coll. (y): age at blood collection in years

^b^The individuals were divided into males (M) and females (F)

^c^Number of individuals with familiar history of PD (Y) and those without any familiar link to the disease (N)

^d^Hoehn and Yahr scale

^e^Total L-Dopa (converted CR + IR) (mg/day): calculated as the controlled release (CR) plus immediate-release (IR) in milligrams per day

The same volume of sample (5 μL) of undepleted plasma was used per patient/control for the SWATH-MS analysis. Additionally, one pool per group (disease versus control group) was prepared by combining part of all the 31 or 28 samples, for PD pool or Ctrl pool, respectively. Five microliters of each pool were used for protein identification and library generation. Before sample processing, the same amount of an internal standard was added to all samples to account for sample loss [13]. The mass spectrometry acquisition and the general data analysis were performed as indicated in the secretome analysis. Still, the quantification was attempted not only for the sample’s specific library (plasma library) but also to the library previously obtained from the secretome analysis (secretome library). Protein levels were estimated based on peptides that met the 1% FDR threshold in at least 1/3 of the samples in a group, and the peak areas of the target fragment ions of those peptides were extracted across the experiment using a 5 min extracted-ion chromatogram (XIC). Protein levels were estimated by summing all the transitions from all the peptides for a given protein (an adaptation of [17]) and further normalized to the levels of the IS.

The comparative analysis of the plasma samples was performed using a Student’s *t*-test using the log_2_ transformed normalized-protein values.

### Informed selection of protein classes as potential biomarkers and evaluation of their biomarker potential

The selection of the mitochondrial-related proteins was performed using the Gene Ontology tool provided at UniProt (https://www.uniprot.org/). Principal component analysis (PCA) was used to evaluate the capacity of the selected candidates (either using all the proteins considered to be altered or only the mitochondrial-related proteins) to distinguish the groups of samples being studied (Ctrl vs. Oxidative stress or PD patients vs. matched controls, for secretome samples or plasma samples, respectively). PCA analysis was performed using the MarkerView software (Ab Sciex®, version 1.2.1) using Pareto scaling [18]. Linear Discriminant Analysis (LDA) and the ROC curve were performed to confirm the capacity of the identified candidates to create a diagnostic model. Correlation analyses were performed to evaluate if the model has potential prognostic value. Spearman’s rank correlation coefficient, Linear Discriminant Analysis (LDA), and ROC curves were performed using IBM® SPSS® Statistics Version 22.

### Independent cohort used for the preliminary validation studies

#### Participants

For the initial validation, 21 patients diagnosed with PD and 12 age-matched controls were gathered (Table 2 and Supplementary Table 2). The study was approved by the Ethics Committee of the Faculty of Medicine of the University of Coimbra (reference CE_010.2017) and the Ethics Committee of the Centro Hospitalar e Universitário de Coimbra (CHUC) (reference 34/CES-CHUC-024-18). It was conducted according to the principles stated in the Declaration of Helsinki [19]. Written informed consent was obtained from all participants. The PD patients were recruited at the Movement Disorders Units of the Neurological Department of the CHUC, where they were assessed by experienced neurologists and were diagnosed according to the criteria defined by the UK Parkinsons’s Disease Society Brain Bank [20].

**Table 2:** Demographic characterization of the cohort used in the preliminary validation

	Age at blood coll. (y)^a^	Gender^b^
**Parkinson’s Disease Group**	60.1 ± 10.7 [38, 79]	M: 9 (40.9%); F: 13 (59.1%)
**Control Group**	67.5 ± 7.2 [53, 75]	M: 3 (25%); F: 9 (75%)

^a^Age at blood coll. (y): age at blood collection in years

^b^The individuals were divided into males (M) and females (F)

All subjects in the present study were selected towards the best age-match between groups and a balanced distribution by gender within each group.

#### ELISA assays

Clusterin, VPS35, and GFP (the internal standard used in mass spectrometry analysis) levels were determined using ELISA commercial kits, namely Clusterin Human ELISA kit (ref.: EHCLU, Invitrogen), Human Vacuolar protein sorting-associated protein 35 (VPS35) ELISA Kit (ref. abx384246, Abbexa) and GFP ELISA Kit (ab171581, Abcam), respectively. All the procedures were performed as indicated by the manufacturer. VPS35 determination was performed using undiluted plasma spiked with GFP, while a 1:50,000 dilution of the samples was used in the case of Clusterin and GFP proteins. In addition, the samples were combined in two pools (Ctrl and PD), and each pool was analyzed in triplicate to evaluate the reproducibility of each method. To achieve a better correlation with the results obtained in the MS analysis, the results were presented as the absorbance of the targets proteins normalized for the absorbance of the IS (GFP).

Methods reproducibility was assessed by analyzing the coefficient of variation of the technical replicates. The statistical analyses were performed using IBM® SPSS® Statistics Version 22 using the Mann–Whitney U test. The level of significance was set at *p* < 0.05. Principal component analysis (PCA) was used to evaluate the capacity of these candidates (either using the raw absorbances or IS-normalized values) to distinguish the two groups. PCA analysis was performed in the MarkerView software (Ab Sciex®, version 1.2.1) using Pareto scaling [18].

## Results

### Cellular model to study the impact of oxidative stress in cells’ secretome

One of the major advantages of translational pipelines for biomarker discovery is the possibility to start the analysis and the candidate selection from well-defined conditions [9]. Considering the key role of oxidative stress in the pathogenesis of neurodegenerative diseases [21], including Parkinson’s Disease (PD), we decided to start by evaluating the alterations induced in the secretome of cells exposed to an oxidative stimulus. Taking into consideration that activation of several signaling pathways, such as those carried out by Mitogen-activated protein kinases (MAPKs) and PI3-Kinase (PI3-K)/Akt pathway, is a common and an important response mechanism to oxidative stress [22–25], the conditions used in this work were defined based on the activation of the ERK1/2 and PI3-K/Akt survival pathways.

**Fig. 1:**
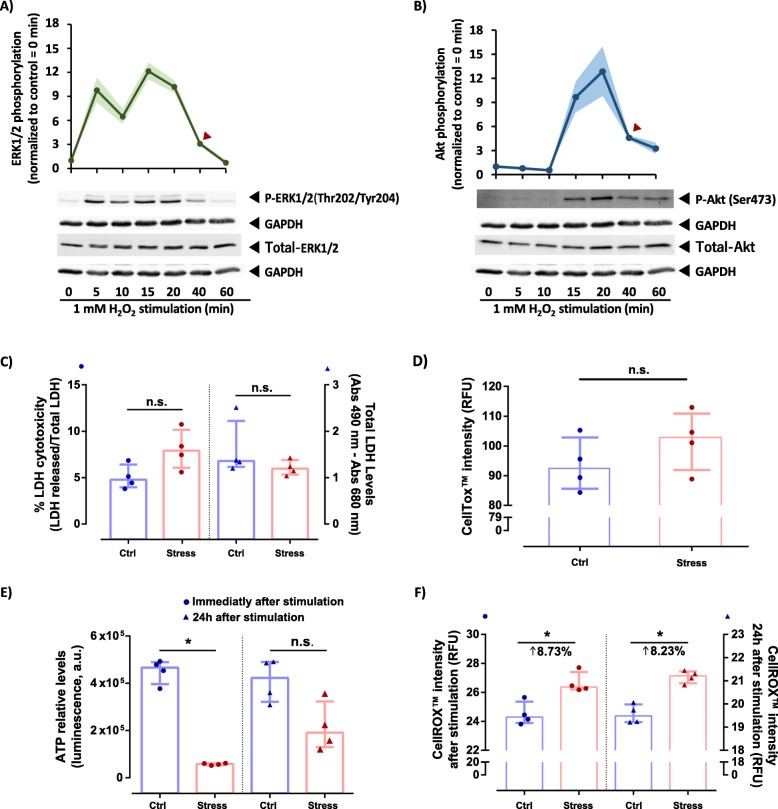
Characterization of the molecular responses of H_2_O_2_-stimulated cells used in the discovery screening. **a-b** Time-course profile for H_2_O_2_-induce ERK 1/2 and Akt activation in HeLa cells. Cells were treated with H_2_O_2_ for the indicated periods, and the activation profiles of ERK1/2 (**a**), and Akt (**b**) were studied by western blot using phospho-specific and anti-total ERK1/2 and Akt antibodies. Anti-GAPDH antibody was used to check protein loading. For each pathway, it is shown a representative western blot (bottom panels) and the profile of activation (top panels) determined by the relative phosphorylation to control conditions (time 0 min). The line indicates the mean, and shadow indicates the SEM of four replicates. Red arrowheads indicate the time-point selected for the differential secretome analysis. **c-f** Biochemical evaluation of HeLa cells response to the proposed oxidative stress condition (1 mM H_2_O_2_ for 40 min). LDH release (**c**), cell toxicity (**d**), ATP levels (**e**), and reactive oxygen species (ROS) levels (**f**) were measured as an indication of the cell integrity, metabolic activity, and oxidative status, respectively. LDH release and cell toxicity (using the CellTox™ Green probe) were measured 24 h after the acute stimuli. LDH cytotoxicity (**c**) represents the relative levels of the released LDH over the total levels of LDH (dots), while the total levels of LDH (triangles) were used as an indication of changes in the number of cells between conditions. ATP and reactive oxygen species levels (measured using the CellROX™ Orange probe) were determined immediately after the 40 min stimulation (dots) and after 24 h (triangles). Bar and error bars correspond to the mean and interquartile range of four experiments, respectively. n.s. and * indicate a *p* ≥ 0.05 and *p* < 0.05, respectively, for statistically significant differences between control (Ctrl, in blue) and oxidative stress (Stress, in red) conditions using the Wilcoxon Signed Rank Test

The activation kinetics were determined by measuring the levels of phosphorylation of ERK 1/2 and Akt (Fig. 1a and b) – respective mediators of the two survival pathways indicated – following the methodology previously described by Ruffels et al. [12]. The activation profiles obtained in this study using HeLa cells were similar to the results previously reported for the SH-SY5Y neuroblastoma cell line [12] – a working model with characteristics identical to dopaminergic neurons, frequently used as a cellular model for the study of PD [26].

The acute stimulation with H_2_O_2_ produced a marked increase in the phosphorylation status of ERK1/2 and Akt (Fig. 1a and b, respectively) reaching the maximal phosphorylation after 15 to 20 min of stimulation, followed by a slight decrease towards levels near the basal state. For this preliminary work and considering the results obtained, a time point after the maximum activation (40 min), in which the phosphorylation levels of these proteins have already declined towards levels similar to the basal activation, was selected to proceed with the secretome analysis. In this sense, it was ensured that the mechanism of response was fully initiated and that cells were primed to respond to oxidative stress.

For secretome analysis, the medium was changed to new media after stimulation, and cells were left in culture for 24 h to collect the molecules secreted by the stimulated cells. To fully characterize the system, several biochemical parameters were assessed (Fig. 1c-f), including: (i) the lactate dehydrogenase release (Fig. 1c) and the staining of dead cells’ DNA (Fig. 1d), indicators of the cell’s membrane permeability; (ii) ATP levels, as an indication of the metabolic state of the cells (Fig. 1e); and iii) a direct measurement of oxidative stress by the use of a reactive oxygen species (ROS) specific fluorogenic probe (Fig. 1f). Taking into account that an acute stimulus for a short period was performed, it is expected that immediately after the stimulation, cells are still actively responding to the insult and, thus metabolically affected. In this sense, to differentiate between the biochemical alterations induced by the active response to the insult from the impairment of cell viability, the ATP levels, and ROS levels were accessed immediately after the 40 min stimulation and after the 24 h period used to collect the secretomes. On the other hand, membrane integrity and cytotoxicity were only evaluated after the 24 h period. In the two tests used to address membrane integrity (Fig. 1c and d), as an indication of cell cytotoxicity, no differences were detected between the control and the stress conditions, indicating that there is no cell death induced by the oxidative stress. The similar levels of total LDH among the two conditions further supported this result, revealing that there was no cell loss when the medium was changed after the 40 min stimulation. On the other hand, the results confirm that cells’ metabolic profile is largely affected in the stress condition when compared with the control: a drastic decrease of the ATP levels immediately after the stimulation period was observed (Fig. 1e), which is partially recovered to levels similar to the control condition after 24 h (no statistical differences were detected at this time point). This severe effect in ATP levels is not accompanied by an increase in cytotoxicity (Fig. 1c and d), and the ATP levels were restored after the 24 h recovery period, confirming that the stimulus performed is not causative of cell death. In this sense, these results may indicate that the reduction in ATP levels may not correspond to a decrease in cell viability, but instead to the increase in oxidative stress and respective cellular response and adaptation. The direct assessment of the ROS levels (Fig. 1f) confirms an increase in 8.7% in the levels of oxidative stress, which were maintained throughout the secretion period. In summary, these results indicate that under the conditions defined for this assay, the cells are characterized by a mild energetic failure associated with an increase in ROS levels [27] but, in general, not associated with an immediate cell death since cells present the capacity to recover from the stimulus after a 24 h recovery period. The observable decrease in ATP levels may result from an increased rate of ATP consumption which is required for a faster response to the acute insult [28], probably combined with a reduction in ATP production associated with mitochondria damage and uncoupling of the respiratory chain, which also contributes to the increase in ROS generation. Similar results were also obtained for SH-SY5Y cells subjected to the same stimuli (data not shown), indicating a similar response between the two systems.

### Secretome analysis and evaluation of mitochondrial-related proteins as potential oxidative stress markers

**Fig. 2:**
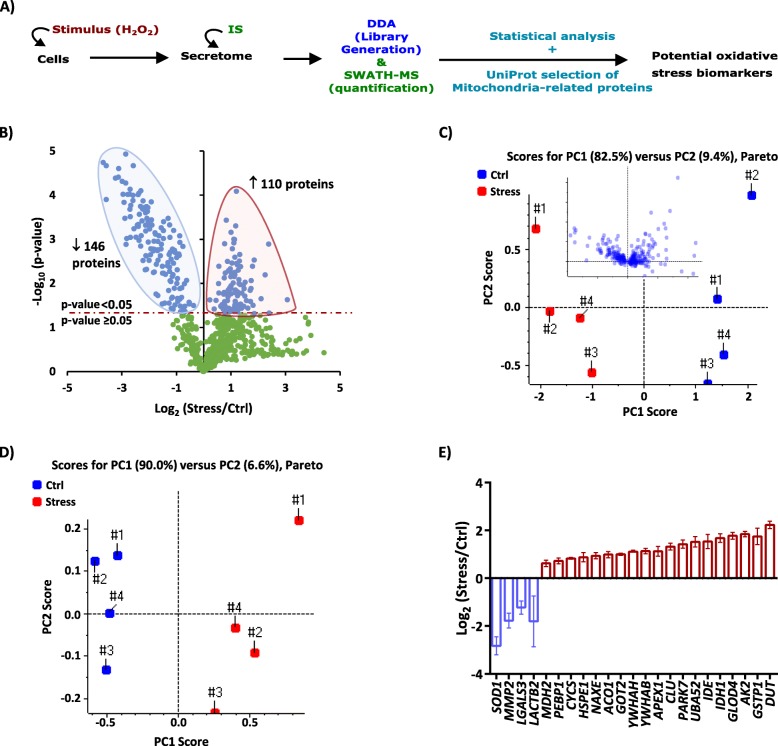
Secretome analysis and identification of mitochondrial-related proteins as potential oxidative stress markers. **a** Schematic representation of the workflow used to identify potential oxidative stress biomarkers from the differential proteomic analysis of the secretome. The selection of candidates was performed by the combined use of statistical analysis and informed selection of groups of proteins based on biological relevance. **b** Volcano plot representing the differential proteome analysis of the secretome between Ctrl and Stress conditions. From the 750 secreted proteins analyzed, a total of 146 proteins were significantly decreased in the oxidative stress condition (blue shadow), and 110 proteins were significantly increased (red shadow). Protein ratios of four replicates were used for statistical analysis. Statistical significance was considered for *p*-values below 0.05 (blue dots) using a One-sample Student’s t-test against a theoretical value of one. **c** Principal component analysis (PCA) using the replicate values of the 146 and 110 proteins significantly decreased and increased in the oxidative stress condition, respectively (**b**). X and Y axis show principal component 1 (PC1) and principal component 2 (PC2) using the Pareto scaling, respectively. The contribution of each component for explaining the total variance is indicated on top of the graphic, suggesting a separation of the conditions across the PC1. The insert plot illustrates the loading distribution. **d** PCA analysis using only the replicate values of 23 mitochondrial-related proteins significantly altered in the oxidative stress condition. The contribution of each principal component for explaining the total variance is indicated on top of the graphic, indicating an improvement in the separation of the experimental conditions in comparison with the use of all the altered proteins (**c**). **e** Graphical representation of the secreted levels of the altered mitochondrial-related proteins. Data correspond to the mean ratio ± SEM of four independent experiments

The secretome analysis (Fig. 2a) reveals a total of 256 proteins altered in the secretome of the cells exposed to the oxidative stress in comparison with the control cells (Fig. 2b and Supplementary Table 3), with 146 proteins decreased, and 110 proteins increased in oxidative stress conditions confirming that cells exposed to a given stimulus altered their extracellular environment in accordance with their intracellular state [9]. Moreover, the results from the secretome analysis demonstrate a good correlation with the results from the biochemical characterization of the cells: there is a tendency to over-secrete redox-related proteins (Supplementary Figure 1), including the protein DJ-1 (Park7) and the cytosolic proteins from the peroxiredoxin family (PRDX1–2 and 6), which is in line with previous reports [29–32]; while there are no alterations in the secretion levels of either two lactate dehydrogenase isoenzymes (LDHA and LDHB) or from a set of nuclear and structural proteins (Histones and Tubulins; Supplementary Figure 1) confirming that there is no significant cell death caused by this oxidative stress conditions.

Thus, the previous results confirm that cells’ secretome can distinguish different cellular states before cell death is observed, being, therefore, an interesting source of circulating biomarkers. Additionally, a “targeted” selection of the candidates was performed by selecting from the statistically significant proteins those belonging to biologically relevant classes (Fig. 2a). In the present study, the focus was the mitochondrial-related proteins, which are known to have key importance in oxidative stress modulation and in the pathogenesis of several neurodegenerative diseases, in particular, Parkinson’s Disease [33–35]. Moreover, as referred above, the results related to the ATP levels seem to indicate some degree of mitochondria malfunction, which further support the importance of analyzing this group of proteins in the present context. In this sense, the list of statistically altered proteins (Fig. 2b), which already promotes good separation of the two experimental conditions using an unsupervised PCA analysis (Fig. 2c), was further filtered considering those classified as mitochondrial-related proteins according to UniProt gene ontology analysis. This selection resulted in a set of 23 proteins that led to an improvement in the separation of the samples from control and oxidative stress conditions (Fig. 2d), confirming the potential of this group of proteins (Fig. 2e) to serve as potential secreted biomarkers of oxidative stress.

### Evaluation of the potential of the proposed strategy for the identification of circulating biomarkers

**Fig. 3:**
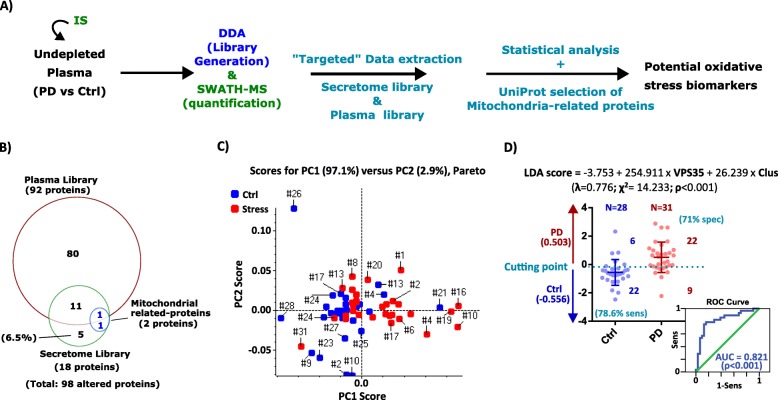
Evaluation of the capacity of the proposed pipeline to identify potential circulating biomarkers. **a** Workflow used to identify potential oxidative stress biomarkers in plasma samples from Parkinson’s Disease (PD) and controls (Ctrl). The workflow used is an adaptation of the pipeline applied to the secretome samples (Fig. 2a) with the addition of a “targeted” data extraction using the spectral library obtained in the secretome analysis to complement the results from the sample-specific library. The selection of candidates was performed by combining statistical analysis and an informed selection of the mitochondrial-related proteins. **b** Venn diagrams comparing the libraries used in the extraction process considering only the 98 altered proteins. The mitochondrial-related proteins found changed are highlighted in blue. **c** PCA analysis using Clusterin and VPS35, two mitochondrial-related proteins involved in apoptotic mechanisms. The contribution of each principal component explaining the total variance is indicated on top of the graphic, indicating some degree of separation of the two groups of samples. **d** Linear discriminant analysis (LDA) using Clusterin and VPS35. The generated LDA discriminant function is indicated on top, with the statistical confidence. The graphic represents the distribution of the samples considering their discriminant score. In the y-axis, it is represented the method decision with the indication of the cutting point (dotted line) and the centroid values (mean values). The error bars indicate the mean value ± SD of 28 and 31 samples from Ctrl and PD groups, respectively. The number of well-classified and misclassified samples are shown on the right side of the plots, as well as the calculated percentages of specificity (spec) and sensitivity (sens). The insert plot illustrates the Receiver operating characteristic (ROC) curve using the discriminant function generated with the two mitochondrial-related proteins. The area under the curve (AUC) value is indicated on the bottom of the image, with the respective statistical confidence

Considering the positive results obtained at the secretome level, the pipeline described above (protein selection based on both statistical parameters and biological importance) was transposed to the analysis of plasma samples (Fig. 3a) as a potential strategy to identify circulating biomarkers. A cohort of 31 PD patients and 28 matched controls were used to test the efficacy of this pipeline at the plasma levels, and in the context of a neurological disease (for which there are only a few cases of translational studies [9]). To that end, 5 μL of undepleted plasma were spiked with an internal standard [13] and analyzed by SWATH-MS as performed for the secretome analysis.

In addition to the conventional analysis in which the quantitative data is extracted from the list of sample-specific identified proteins, in the present pipeline, the quantification was also attempted considering the list of proteins identified in the secretome analysis. This combined analysis allowed the quantification of a total of 196 proteins (Supplementary Figure 2A and Supplementary Table 4) from which the information of 14 proteins was only available at the secretome library, representing an increase of almost 8% in the number of quantified proteins. From these proteins, 98 proteins (Fig. 3b) were significantly altered between PD patients and controls, with a clear tendency to be increased in PD samples (89 increased proteins versus 9 proteins decreased in PD plasma samples – Supplementary Figure 2B). Again, the use of the secretome library was beneficial, allowing the identification of 6 significantly altered proteins that would have been lost if only a conventional analysis had been performed (Fig. 3b), which corresponds to an increase of 6.5%. Importantly, from the two mitochondrial-related proteins identified from the list of altered proteins – Clusterin and the Vacuolar protein sorting-associated protein 35 (VPS35) –, the latter was only included in the analysis because it was previously found in the secretome screening (Fig. 3b).

Interestingly, these two proteins were already associated with PD and other neurodegenerative diseases. Clusterin has been indicated as a potential plasma or cerebrospinal fluid (CSF) biomarker of both PD and Alzheimer’s disease (AD), however, with controversial and non-reproducible results [36–42]. On the other hand, VPS35 is one of the PD-linked products (also known as Parkinson’s disease 17 - PARK17) and the third autosomal-dominant gene associated with PD [43, 44]. VPS35 is an essential component of the retromer complex, being involved in the endosome-to-Golgi and endosome–to–plasma membrane retrieval of membrane proteins and indicated as a critical player in the control of mitochondrial turnover [45–47]. Contrary to the protein Clusterin, this protein was never identified in plasma samples. Both proteins are involved in apoptotic mitochondrial changes [47, 48]; thus, the combination of these two proteins may result in a potential indicator of cell death transversal to other neurodegenerative diseases.

Contrary to what was observed in the case of the secretome analysis, the use of all the 98 altered proteins does not result in a good group separation (Supplementary Figure 2C); on the other hand, the combination of only these two mitochondrial-related proteins reveals some potential to separate the PD from Ctrl samples (Fig. 3c). This potential was further confirmed by the capacity to originate a Linear Discriminate model (Fig. 2d) with potential diagnostic value to discriminate PD patients from age- and gender-matched-controls (*p*-value< 0.001). Specifically, this analysis originates a model with a specificity of 71% and a sensitivity of 78.6%. With the application of this model, it was possible to correctly classify 74.6% of the total number of samples and 72.9% of the samples in a cross-validation study (Supplementary Figure 3). Finally, the ROC analysis presented an average area under the curve (AUC) of 0.821 (95% confidence interval: 0.71–0.93, p-value< 0.001), revealing a good diagnostic potential [49]. Importantly, this model shows to be more reliable than the individual results of each protein (Supplementary Figure 4), demonstrating the importance of having a panel of candidates rather than a single biomarker.

### Evaluation of the prognostic potential of the identified candidates and preliminary validation in an independent cohort

Considering the potential revealed by these two proteins, their capacity to distinguish different disease states (their prognostic potential) was further investigated. The limited number of PD patients used in this pilot work (31 in total), precluded the possibility to generate an LDA discriminant function specific for the discrimination between PD patients since each group would have only a few samples. In addition, the PD cohort under study is mainly composed of patients with scores below 2 in the Hoehn and Yahr (HY) scale (Supplementary Table 1), thus lacking a proper distribution considering different disease states. Hence, the disease duration was used as an indirect indication of the progression of the disease [50], and the PD cohort was subdivided into three groups as follows: patients with less than 5 years of disease (12 individuals), patients with 5 to 9 years of disease (11 patients) and patients with more than 9 years of disease (8 patients).

**Fig. 4:**
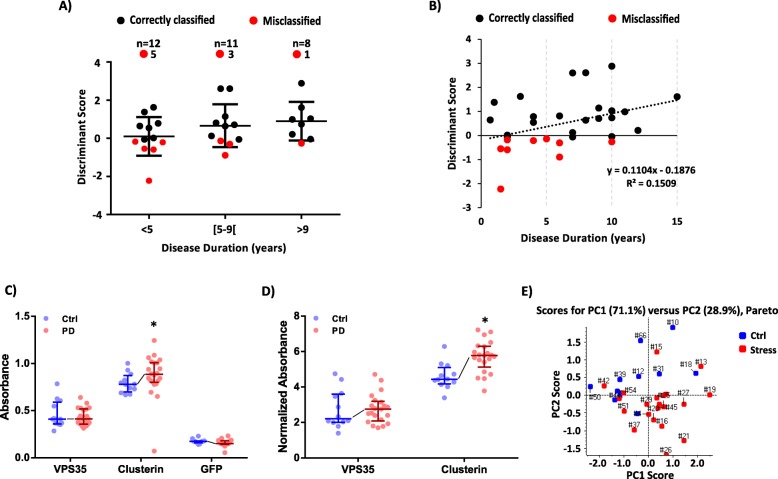
Evaluation of the prognostic potential of the candidates identified and preliminary validation. **a** Distribution of the patients’ discriminant scores according to the disease duration. Patients were grouped into 3 classes (until 5 years, 5 to 9 years, and more than 9 years of disease duration). The number of patients in each group is indicated at the top of the graphic, as well as, the number of misclassified cases (red markers). **b** Scatter-plot of the discriminant scores considering the disease duration, with the respective trend line (dot-line), equation, and coefficient of determination (R^2^). The red dots correspond to misclassified cases. Discriminant scores (patients classification) present a low but significant correlation with the disease duration (Spearman’s Rank correlation coefficient (r) of 0.388 and a *p*-value of 0.031). (**c**-**d**) ELISA results using an independent cohort of samples. **c** Distribution of the absolute absorbances of GFP, Clusterin, and VPS35 proteins measured in each sample. The graphic indicates the median value ± interquartile range of 12 and 21 samples from Ctrl and PD groups, respectively. The line represents the median tendency between groups. *, *p* < 0.05, indicates significant differences for the control condition using the Mann–Whitney U test. **d** Distribution of the Clusterin and VPS35 normalized absorbances (protein absorbance normalized to the IS absorbance). The graphic indicates the median value ± interquartile range of 12 and 21 samples from Ctrl and PD groups, respectively. The line represents the median tendency between groups. *, *p* < 0.05, indicates significant differences for the control condition using the Mann–Whitney U test. **e** Principal component analysis using the normalized absorbance values of Clusterin and VPS35. The contribution of each principal component for explaining the total variance is indicated on top of the graphic. PCA processing was performed using the Pareto scaling

By distributing the individual discriminant scores within the three groups (Fig. 4a), it was possible to observe a mean tendency to have a gradual increase in the discriminant score (which indicates a more robust positive classification) as the duration of the disease increases in approximately 5 year-intervals. Moreover, it was also possible to observe an opposite tendency regarding the number of misclassified individuals (i.e., the number of misclassification, indicated in red, decreases with the increase in the disease duration). To further characterize this relation, a correlation analysis was done (Fig. 4b), which points out for a weak (r = 0.388) but statistically significant (*p*-value of 0.031) positive correlation between the disease duration and the discriminant scores. This, ultimately indicates that there is a tendency to have higher circulating levels of mitochondrial-related proteins with the progression of the disease. Although only a weak correlation was observed, this association was more evident than the correlation between the disease duration and the HY scores (Supplementary Figure 5), which reveals not to be correlated in the present cohort (r = 0.262 and a p-value of 0.154).

Finally, to further evaluate the capacity to move this pipeline to clinical practice, a preliminary validation in an independent cohort and using commercially available ELISA kits was performed. To better correlate the two types of analysis (MS and ELISA), the conventional ELISA pipeline was adapted to be more in line with the MS data processing. Thus, the samples were spiked with the same amount of the IS used in the MS analysis (MBP-GFP), and the IS (the protein GFP) was also quantified by ELISA (Supplementary Figure 6A and Fig. 4c) in addition to the analysis of two candidates (VPS35 and Clusterin). Moreover, instead of determining the concentration of each protein, the analysis was done using the absorbance values, which were further normalized to the values of the IS, as performed in the MS analysis. While a clear increase in the Clusterin levels was observed in PD patients using both non-normalized (Fig. 4c) and normalized data (Fig. 4d), the same was not observed in the case of the protein VPS35. A small and non-significant increase in VPS35 was only observed after normalization of the values. The lack of significance in the VPS35 results may be a combination of a reduced number of samples and the lack of other commercially available kits. Nevertheless, the present results already revealed a tendency to be increased in PD samples.

The reduced number of patients and the lack of statistical significance in the case of VPS35 preclude the hypothesis of building a diagnostic model. Nevertheless, the PCA analysis using the combination of the ELISA results of these two proteins reveals their capacity to promote a good separation of the Ctrl and PD patients, even using a cohort different from the one used in the discovery phase (Fig. 4e). The results also showed that the use of normalized data improves the capacity to separate the groups (Supplementary Figure 6B for the PCA analysis with the no-normalized values), demonstrating the importance of adapting the pipelines used in discovery and validation studies to be performed in a more similar way.

## Discussion

The present work indicates that a translational pipeline for biomarker discovery, in the present case the use of cells’ secretomes obtained under well-defined condition in combination with an informed selection of the biomarker candidates based on relevant biological functions (as it was recently proposed [10]), might be an interesting and complementary strategy to identify potential circulating biomarkers for neurological diseases, in this case, Parkinson’s disease.

In fact, despite several years of research focused on the identification of potential molecular biomarkers of neurodegenerative diseases, there is no biochemical diagnostic model that helps in the diagnosis of those diseases, including PD [51]. Moreover, critical reviews regarding the potential candidates highlighted so far, have reduced the list of best candidates to a few molecules that have already a known link to the PD pathogenesis and other neurodegenerative diseases, such as the protein DJ-1, oligomerized α-synuclein, tau, phospho-tau, amyloid β1–42 [for concise reviews on this topic consult [5, 6, 51]]. This evidence further confirms the importance of including the biological relevance as a selection criterion in the biomarker discovery pipelines, as it was introduced in this work.

On top of that, the use of the SWATH-MS approach also brings some advantages to this translational workflow. In fact, even in the majority of the translational works that start with the use of secretomes in the discovery phase, there is a tendency to select only some few candidates to measure in a targeted way in the human samples [9]. The use of SWATH-MS allows the combination of a “targeted” analysis of the previously identify candidates, with the conventional untargeted analysis of the samples from the disease cohorts, thus allowing the continuous addition of more data, which increases the probability to identify the best candidates. Additionally, since SWATH-MS allows a complete record of the samples, the files acquired in a given experiment can be further re-analyzed in combination with new acquisitions; therefore, allowing to increase the size of the cohorts without the need of re-analyzing the samples again, also facilitating retrospective assays [11].

Moreover, for being a selection method that is not focused on highlighting the differences between the individuals from the groups used in the discovery study, the candidates identified via this pipeline may have better chances to be transposed to the overall population. Concurrently, this may also be a good method to avoid the confounding factors characteristic of this type of patients (elderly people that usually have other comorbidities).

Although only two-proteins were highlighted in the present work, the results obtained already points for the possibility of mitochondrial-associated proteins as an interesting class with diagnostic potential for neurodegenerative diseases like PD. Moreover, the pieces of evidence observed in this pilot study also emphasize the importance of combining several proteins for building a robust diagnostic model (Supplementary Figure 4), which may have more chances to be successful in the clinical practice.

Additional validation studies using different cohorts and/or different analytical techniques should be performed before confirming the biomarker potential of the two proteins identified in this pilot study. In addition to that, further studies should also be performed to try to include more proteins from the same class or other relevant classes to strengthen the diagnostic capacity of the model. In fact, this continuous improvement of the model can be easily achieved by following the pipeline presented in this work, since it was settled on the use of SWATH-MS.

## Conclusion

In the present work, an adaptation of a translational pipeline for biomarker selection was presented and transposed to neurological diseases. From the application of this adapted pipeline, two mitochondrial-related proteins were identified as potential candidates for Parkinson’s disease diagnosis. The novelty and success of this pilot study may arise from the combination of: i) a translational research pipeline, where plasma samples are interrogated using the knowledge previously obtained from cells’ secretome under oxidative stress; ii) the combined use of statistical analysis and an informed candidate selection based on relevant disease mechanisms, and iii) the use of SWATH-MS, an untargeted MS method that allows a complete record of the acquired samples and a targeted data extraction of the information previously acquired.

## Supplementary information

**Additional file 1: Supplementary Methods.** Document containing: 1) detailed information on the methods used in the present work.

**Additional file 2: Supplementary Figures.** Document containing the following supporting images.

**Additional file 3: Supplementary Table 1.** Data of patients and control individuals included in the discovery study.

**Additional file 4: Supplementary Table 2.** Data of patients and control individuals used in the preliminary validation.

**Additional file 5: Supplementary Table 3.** Evaluation of the total levels of the secreted proteins under control and stress conditions.

**Additional file 6: Supplementary Table 4.** Evaluation of the total levels of the plasma proteins of Controls and Parkinson’s disease patients.

## Data Availability

The mass spectrometry proteomics data from secretome analysis have been deposited to the ProteomeXchange Consortium via the PRIDE [52] partner repository with the dataset identifier PXD009068”. The results of both experiments can be found at Supplementary Tables 3 (for secretome analysis) and 4 (for plasma samples) supplied in separate.

## Abbreviations

AD: Alzheimer’s Disease
CHCB: Centro Hospitalar Cova Da Beira
Ctrl: Control
FDR: False Discovery Rate
IDA: Information-Dependent Acquisition
LDA: Linear Discriminant Analysis
LDH: Lactate Dehydrogenase
MAPKs: Mitogen-Activated Protein Kinases
PCA: Principal Component Analysis
PD: Parkinson’s Disease
ROC: Receiver Operating Characteristic
ROS: Reactive Oxygen Species
SWATH-MS: Sequential Window Acquisition Of All Theoretical Mass Spectra
UKPDBBC: Uk Parkinson’s Disease Society Brain Bank Clinical Diagnostic Criteria
XIC: Extracted-Ion Chromatogram
