# Supplementary material for: A different vision of translational research in biomarker discovery: a pilot study on circulatory mitochondrial proteins as Parkinson’s disease potential biomarkers
Source: Transl Neurodegener. 2020 Apr 3;9:11. doi: 10.1186/s40035-020-00188-0 (PMC7118951; doi:10.1186/s40035-020-00188-0)
Supplement: Supplementary file 1 — Additional file 1: Supplementary Methods. Document containing: 1) detailed information on the methods used in the present work. [file 40035_2020_188_MOESM1_ESM.pdf]

Supplementary Methods

1 Detailed MethodsTime Course Activation of ERK1/2 and Akt

The activation of ERK1/2 and Akt was performed based on the previous work of Ruffels *et al.* (2004), where the activation of different kinases in SH-SY5Y cells was induced by hydrogen peroxide (H_2O_2). HeLa cells were grown (at 37 °C, 95 % air and 5 % CO_2 in humidified incubator) in DMEM medium supplemented with 10 % FBS, Pen-Strep, and Amphotericin B. Pathways activation was verified by immunoblot against the phosphorylated forms of ERK1/2 (P-ERK 1/2) and Akt (P-Akt), the total amount of ERK1/2 and Akt, and the GPDH to control the protein levels (loading control). The procedure was similar to the one used in the work of Ruffels *et al.* (2004) with some modifications.

1.1.1 *Redox Stimuli*

HeLa cells were seeded at 12×10^3 cells/cm^2 in 6-well plates (Corning). After 24 hours, serum starvation was induced by changing the culture medium to DMEM medium containing no FBS. Cells were maintained in the serum-reduced medium for 16 h. Serum-starved cells were then treated with 1 mM of H_2O_2 during different times points tested: 0, 5, 10, 15, 20, 40, and 60 minutes. Stimulation was stopped by aspiration of the medium followed by a washing step with pre-warmed PBS and addition of ice-cold lysis buffer [RIPA buffer (50 mM Tris-HCl, pH 7.4; 1 % (v/v) Igepal; 0.25 % (v/v) sodium-deoxycholate; 150 mM NaCl; 1 mM DTT; 1 mM EDTA, Complete Mini protease inhibitor mixture and Complete Mini phosphatase inhibitor mixture)].

1.1.2 *Preparation of Protein Extracts*

After the addition of RIPA buffer, cell culture plates were placed on ice and the lysates were obtained by scraping the plate with a rubber cells scraper. The lysates were collected to microcentrifuge tubes and protein extraction was promoted by sonication (30 seconds (s) with 40% amplitude of cycles of 1 s), followed by removal of insoluble fraction via centrifugation at 20,000×g for 15 min at 4 °C. After centrifugation, the supernatant (protein extract) was collected into new tubes. A sample of each protein extract was used to calculate the protein concentration

using 2-D Quant kit (GE Healthcare) according to the manufacturer's instructions, and the remaining extracts were stored at -20°C until further use.

1.1.3 *Immunoblotting Detection*

The correspondent volume of the 6× SDS Sample Buffer [(0.35 M solution of Tris-HCl with 0.4 % SDS (v/v), pH 6.8, 30 % glycerol (v/v), 10 % SDS (w/v), 9.3 % DTT (w/v) and 0.01 % bromophenol blue (w/v)] was added to the protein extracts to a 1× final concentration. The proteins were then denatured by boiling at 95 °C for 5 min. Equal amounts of protein per lane (25 µg of total protein extract) were electrophoretically separated on 12.5 % SDS-polyacrylamide gels using a mini-PROTEAN® Tetra Electrophoresis System (Bio-Rad). Each sample was loaded in two gels, one of them used to detect the phosphorylated forms and the other the total levels of ERK1/2 and Akt. Proteins were transferred to low fluorescence polyvinylidene fluoride (PVDF) membranes (TBT RTA TRANSFER KIT, Bio-Rad) using a Trans-Blot Turbo Transfer System (BioRad) during 30 min at a constant voltage of 25 V (with the amperage limited to 1 A). Following transfer, the membranes were blocked for 1 h at room temperature (RT) with 5 % (w/v) skimmed milk powder in PBS-Tween 20 (PBS-T) [0.1 % (v/v)]. Blots were then incubated overnight at 4 °C followed by 2 h at RT with primary antibodies, according to Table 1, prepared in the blocking solution. Primary antibodies were removed, and membranes were extensively washed with PBS-T (3 times, 15 min under agitation each time). Blots were then incubated for 2 h at RT with the respective secondary antibody conjugated with alkaline phosphatase (anti-mouse (1:10000; ref.: 115-055-003) or anti-rabbit (1:3000; ref.: 111-055-003) Jackson ImmunoResearch Laboratories, Inc.) in 5 % (w/v) skimmed milk powder dissolved in PBS-T followed by extensive washes as above. Membranes were first incubated with the phospho- or total-specific antibodies followed by re-probing of the membranes with antibody against the GAPDH.

Protein-immunoreactive bands were developed using the "Enhanced Chemifluorescence (ECF) detection system" (GE Healthcare) and visualized in a Molecular Imager FX System (Bio-Rad).

Table 1 - List of primary antibodies used in Western blot analysis of ERK1/2 and Akt activation.

	MW (kDa)	Species	Dilution	Source (ref.)
**Anti-P-Akt (Ser473)**	60	Rabbit	1:500	Cell Signaling Technology, Inc. (#9271)
**Anti-total Akt**	60	Rabbit	1:500	Cell Signaling Technology, Inc. (#9272)
**Anti-P-ERK1/2 (Thr202/Tyr204)**	42/44	Rabbit	1:500	Cell Signaling Technology, Inc. (#4377)
**Anti-total ERK1/2**	42/44	Mouse	1:500	Cell Signaling Technology, Inc. (#9107)
**Anti-GFP**	37	Mouse	1:250	Santa Cruz Biotechnology, Inc. (sc-47724)

MW: Molecular weight; **Ser:** serine; **Thr**: threonine; **Tyr**: tyrosine.

1.1.4 *Data analysis*

The adjusted volumes (total intensities in a given area with local background subtraction) for each band were obtained using Image Lab software (version 5.1, Bio-Rad). All bands were adjusted to the loading control (GAPDH), and the phosphorylation levels were further adjusted to the respective total levels. To trace the profile of activation of ERK1/2 and Akt pathways during the time course proposed, the adjusted phosphorylation levels were normalized to the control condition (0 min H_2O_2 stimulation). Data were presented as mean ± standard error of the mean (S.E.M.), and every experimental condition was tested in four sets of independent experiments.

1.2 Evaluation of cell toxicity, metabolic state, and oxidative stress

Different parameters were also assessed to characterize the impact of oxidative stress caused by the selected condition (40 min stimulation with 1 mM H_2O_2). Cell Titer Glo® Luminescent Cell Viability Assay (Promega) was used to measure the metabolically active cells by quantitation of cellular ATP levels, membrane integrity (cytolysis) and cytotoxicity were evaluated by Pierce Lactate dehydrogenase (LDH) Cytotoxicity assay kit (Thermo Scientific) to measure LDH

released from damaged cells and by CellTox™ Green Cytotoxicity Assay (Promega) which stains the dead cells' DNA, and finally, oxidative stress detection was performed by measuring the relative oxygen species (ROS) using CellROX® Orange Reagent (Invitrogen).

1.2.1 *Redox Stimuli*

HeLa cells were seeded at 12,000 cells/cm^2 in DMEM medium with 10 % FBS in 96-well plates (Corning) at 37°C, 5 % CO2/95 % air in a humidified incubator. Twenty-four hours after plating, the culture medium was changed to DMEM medium with no FBS and cells were serum-starved for 16 h before stimulation. After starvation, cells were stimulated with a freshly made solution of 1 mM of H_2O_2 in DMEM for 0 or 40 min.

ATP and ROS levels were assessed immediately after the stimulation and after 24h after stimulation. For the 24 h measurement, the medium was changed to a new DMEM medium without FBS after the stimulation and cells were allowed to recover for 24 h. Each condition was performed in a total of four replicates.

1.2.2 *Cell titer Glo®*

The experiments to evaluate the ATP levels were performed in white opaque 96-well plates (Corning) using the Cell Titer-Glo® Luminescent assay (Promega) according to the manufacture's indications. Briefly, after stimulation or after stimulation followed by the 24 h recovery period the CellTitter Glo® reagent was added to each well in a 1:1 proportion relative to the culture medium, then the plate was agitated for 2 min at 300 rpm to promote cell lysis followed by an incubation of 15 min, in the dark, to stabilize the luminescent signal. The luminescent signal was detected by a LUMIstar Galaxy automated microplate luminescence reader (BMG Labtech), according to the manufacturer's instructions. This method determines the metabolic active cells based on bioluminescent quantitation of the ATP present in the sample, which indicates the presence of metabolically active cells.

1.2.3 *LDH cytotoxicity assay*

LDH release was measured to evaluate the cellular membrane permeability. HeLa cells were plated in 96-well plates for the analysis after the 24 h recovery period. For each time point analyzed (0

and 40 min) it was accessed the total amount of LDH (as an indication of the total number of cells) and the LDH spontaneously released (an indicator of membrane permeability). Briefly, after stimulation or the recovery period, 25 µL of the culture medium were transferred to a 384-well plate to access the spontaneous release of LDH and 1 µL of Triton X-100 was added to the cells to promote cell lysis after which 25 µL of medium were transferred to the 384-well plate (to monitoring the total LDH). Twenty-five microliters of the Reaction Mixture provided in the Pierce LDH Cytotoxicity Assay Kit were added to each sample (the 25 µL medium collected to the 384-well plate) followed by an incubation period of 30 min at RT. The reaction was stopped by addition of 25 µL of the Stop Solution provided in the kit and the absorbance was measured at 490 nm and 680 nm (background signal) in a Microplate Spectrophotometer (PowerWave XS, BioTek). The background signal (680 nm) was automatically subtracted from the 490 nm absorbance, and the percentage of cytotoxicity (% Cytotoxicity) was determined for each condition by calculating the ratio of the LDH released to the total LDH.

1.2.4 *CellTox™ Green Cytotoxicity Assay*

The experiments were performed in white opaque 96-well plates (Corning) according to the manufacture's indications. Briefly, 24 h after the stimulation CellTox™ Green reagent was added to each well in a 1:1 proportion relative to the culture medium, then the plate was agitated for 1 min at 300 rpm to ensure homogeneity followed by incubation at room temperature of 15 min, in the dark, to stabilize the signal. The fluorescent signal was detected in a Gemini EM Fluorescence Microplate Reader (Molecular Devices, LLC) with excitation and emission wavelengths of 485 and 530 nm, respectively.

1.2.5 *CellRox® Oxidative Stress Reagent (Orange)*

The evaluation of the oxidative stress was performed in black opaque 96-well plates (Corning) using the CellRox® Orange Reagent (Invitrogen). Briefly, after stimulation, the CellRox® Orange Reagent was added to each well to a final concentration of 5 µM followed by incubation for 10 min at 37 °C. The fluorescent signal was detected in a Gemini EM Fluorescence Microplate Reader (Molecular Devices, LLC) with excitation and emission wavelengths of 485 and 555 nm, respectively.

1.2.6 *Data analysis*

All the statistical analyses were performed using IBM® SPSS® Statistics Version 22. Single comparisons between the different conditions studied were made using Wilcoxon Signed Rank test. The level of significance in all the statistical analyses was set at $p<0.05$.

1.3 Mass spectrometry analysis by SWATH mode

Both experiments (secretomes and plasma samples) were analyzed on an AB Sciex 5600 TripleTOF in two modes: information-dependent acquisition (IDA) of the pooled samples for protein identification and library generation, and SWATH acquisition of individual replicates for quantitative analysis. Peptides separation was performed using liquid chromatography (nanoLC Ultra 2D, Eksigent®) on a ChromXP™ C18CL reverse phase column (300 µm × 15 cm, 3 µm, 120 Å, Eksigent®) at 5 µL/min. The gradients used were slightly different among the two experiments to accommodate the introduction of DMSO in the mobile phases: i) for secretome analysis it was used the following multistep gradient 0-2 min linear gradient from 5 to 10 %, 2-45 min linear gradient from 10 % to 30 %, and 45-46 min to 35 % of acetonitrile in 0.1 % formic acid (FA); in the case of the plasma samples the gradient used was the following 0-2 min linear gradient from 2 to 5 %, 2-45 min linear gradient from 5 % to 30 %, and 45-46 min to 35 % of acetonitrile in 0.1 % FA and 5% DMSO. Peptides were eluted into the mass spectrometer using an electrospray ionization source (DuoSpray™ Source, AB Sciex) with a 50 µm internal diameter (ID) stainless steel emitter (New Objective).

For IDA experiments, the mass spectrometer was set to scanning full spectra (350-1250 m/z) for 250 ms, followed by up to 100 MS/MS scans (100–1500 m/z from a dynamic accumulation time – minimum 30 ms for precursor above the intensity threshold of 1000 – in order to maintain a cycle time of 3.3 s). Candidate ions with a charge state between +2 and +5 and counts above a minimum threshold of 10 counts per second were isolated for fragmentation and one MS/MS spectrum was collected before adding those ions to the exclusion list for 25 seconds (mass spectrometer operated by Analyst® TF 1.7, AB Sciex®). The rolling collision was used with a collision energy spread of 5.

For the SWATH-MS experiments, a set of 60 windows of variable width (containing 1 *m/z* for the window overlap, Table 2) was constructed covering the precursor mass range of 350-1250 *m/z*. A

200 ms survey scan (350-1500 *m/z*) was acquired at the beginning of each cycle for instrument calibration and SWATH-MS/MS spectra were collected from 100–1500 *m/z* for 50 ms resulting in a cycle time of 3.25 s from the precursors ranging from 350 to 1250 *m/z*, which is compatible with the acquisition of at least 8 points per chromatographic peak. The collision energy for each window was determined according to the calculation for a charge +2 ion centered upon the window with a collision energy spread of 15.

Table 2 - SWATH-MS variable windows. For each window, it is indicated the *m/z* range and the window width in Dalton (Da).

	m/z **range**	**Width (Da)**
**Window 1**	349.5-360.9	11.4
**Window 2**	359.9-375.2	15.3
**Window 3**	374.2-389.2	15
**Window 4**	388.2-402.2	14
**Window 5**	401.2-415.3	14.1
**Window 6**	414.3-427.4	13.1
**Window 7**	426.4-439.1	12.7
**Window 8**	438.1-449.9	11.8
**Window 9**	448.9-460.7	11.8
**Window 10**	459.7-471.1	11.4
**Window 11**	470.1-480.5	10.4
**Window 12**	479.5-490	10.5
**Window 13**	489-499	10
**Window 14**	498-508	10
**Window 15**	507-516.5	9.5
**Window 16**	515.5-525.1	9.6
**Window 17**	524.1-533.2	9.1
**Window 18**	532.2-540.8	8.6

**Window 19**	539.8-548.5	8.7
**Window 20**	547.5-555.7	8.2
**Window 21**	554.7-563.4	8.7
**Window 22**	562.4-570.6	8.2
**Window 23**	569.6-577.8	8.2
**Window 24**	576.8-585.4	8.6
**Window 25**	584.4-592.6	8.2
**Window 26**	591.6-600.3	8.7
**Window 27**	599.3-607.9	8.6
**Window 28**	606.9-615.6	8.7
**Window 29**	614.6-623.2	8.6
**Window 30**	622.2-630.9	8.7
**Window 31**	629.9-638.5	8.6
**Window 32**	637.5-646.2	8.7
**Window 33**	645.2-653.8	8.6
**Window 34**	652.8-661.5	8.7
**Window 35**	660.5-669.1	8.6
**Window 36**	668.1-677.2	9.1
**Window 37**	676.2-685.3	9.1
**Window 38**	684.3-693.9	9.6
**Window 39**	692.9-702.9	10
**Window 40**	701.9-711.9	10
**Window 41**	710.9-721.3	10.4
**Window 42**	720.3-731.2	10.9
**Window 43**	730.2-741.6	11.4
**Window 44**	740.6-752.4	11.8
**Window 45**	751.4-763.6	12.2
**Window 46**	762.6-775.8	13.2
**Window 47**	774.8-787.9	13.1
**Window 48**	786.9-800.5	13.6
**Window 49**	799.5-814.5	15
**Window 50**	813.5-829.3	15.8

**Window 51**	828.3-845.5	17.2
**Window 52**	844.5-865.3	20.8
**Window 53**	864.3-886.5	22.2
**Window 54**	885.5-911.2	25.7
**Window 55**	910.2-939.1	28.9
**Window 56**	938.1-972	33.9
**Window 57**	971-1008.4	37.4
**Window 58**	1007.4-1053.4	46
**Window 59**	1052.4-1120	67.6
**Window 60**	1119-1249.6	130.6

Protein identification was obtained using ProteinPilot™ software (v5.0, AB Sciex®). For each experiment the respective IDA files were combined in a single search against a database composed by the *Homo sapiens* database from SwissProt (released at August 2014 or June 2017, for secretome and plasma analysis, respectively) and the sequences of the recombinant protein used as internal standard (MBP-GFP (Anjo et al., 2018)), using trypsin and acrylamide as alkylating agent, and a special focus option for gel-based approaches. In the case of the plasma samples, positive identifications were considered when identified proteins and peptides reached a 5 % local FDR (Tang et al., 2008;Sennels et al., 2009). The identification files were used as libraries of precursor masses and fragment ions for subsequent SWATH processing.

SWATH data was processed in the SWATH™ processing plug-in for PeakView™ (v2.0.01, AB SCIEX). Briefly, the chromatographic profiles of the peptides presented in the libraries were extracted from the SWATH-MS data for up to 5 target fragment ions of up to 15 peptides per protein (peptides and target fragment ions were selected automatically from the library using the criteria described in (Anjo et al., 2015)). Additionally, the retention time was adjusted to each sample using the IS peptides.

Peptide features that met the 1% FDR threshold in all the replicates (for IS analysis), in at least 3 biological replicates (for the secretome analysis) or at least 1/3 of the samples per group (in the case of the PD vs controls) were retained, and the peak areas of the target fragment ions of those peptides were extracted across the experiment using a 4-5 min extracted-ion chromatogram (XIC)

window adjusted in order to accommodate the entire chromatographic peaks. Protein levels were estimated by summing all the transitions from all the peptides of a particular protein (Anjo et al., 2015) and normalized to the levels of the internal standard (Anjo et al., 2018).

The mass spectrometry proteomics data from secretome analysis have been deposited to the ProteomeXchange Consortium via the PRIDE (Vizcaino et al., 2016) partner repository with the dataset identifier PXD009068". The results of both experiments can be found at Supplementary Tables 1 (for secretome analysis) and 2 (for plasma samples) supplied in separate.

References:

Anjo, S.I., Santa, C., and Manadas, B. (2015). Short GeLC-SWATH: a fast and reliable quantitative approach for proteomic screenings. *Proteomics* 15**,** 757-762.

Anjo, S.I., Simões, I., Castanheira, P., Grãos, M., and Manadas, B. (2018). A generic normalization method for proper quantification in untargeted proteomics screening. *bioRxiv*.

Association, G.a.O.T.W.M. (2014). World Medical Association Declaration of Helsinki: ethical principles for medical research involving human subjects. *The Journal of the American College of Dentists* 81**,** 14.

Hughes, A.J., Daniel, S.E., Kilford, L., and Lees, A.J. (1992). Accuracy of clinical diagnosis of idiopathic Parkinson's disease: a clinico-pathological study of 100 cases. *Journal of Neurology, Neurosurgery & Psychiatry* 55**,** 181-184.

Ruffels, J., Griffin, M., and Dickenson, J.M. (2004). Activation of ERK1/2, JNK and PKB by hydrogen peroxide in human SH-SY5Y neuroblastoma cells: role of ERK1/2 in H2O2-induced cell death. *Eur J Pharmacol* 483**,** 163-173.

Sennels, L., Bukowski-Wills, J.C., and Rappsilber, J. (2009). Improved results in proteomics by use of local and peptide-class specific false discovery rates. *BMC Bioinformatics* 10**,** 179.

Tang, W.H., Shilov, I.V., and Seymour, S.L. (2008). Nonlinear fitting method for determining local false discovery rates from decoy database searches. *J Proteome Res* 7**,** 3661-3667.

Van Den Berg, R.A., Hoefsloot, H.C., Westerhuis, J.A., Smilde, A.K., and Van Der Werf, M.J. (2006). Centering, scaling, and transformations: improving the biological information content of metabolomics data. *BMC genomics* 7**,** 142.

Vizcaino, J.A., Csordas, A., Del-Toro, N., Dianes, J.A., Griss, J., Lavidas, I., Mayer, G., Perez-Riverol, Y., Reisinger, F., Ternent, T., Xu, Q.W., Wang, R., and Hermjakob, H. (2016). 2016 update of the PRIDE database and its related tools. *Nucleic Acids Res* 44**,** 11033.
