# Supplementary material for: A different vision of translational research in biomarker discovery: a pilot study on circulatory mitochondrial proteins as Parkinson’s disease potential biomarkers
Source: Transl Neurodegener. 2020 Apr 3;9:11. doi: 10.1186/s40035-020-00188-0 (PMC7118951; doi:10.1186/s40035-020-00188-0)
Supplement: Supplementary file 2 — Additional file 2: Supplementary Figures. Document containing the following supporting images. [file 40035_2020_188_MOESM2_ESM.pdf]

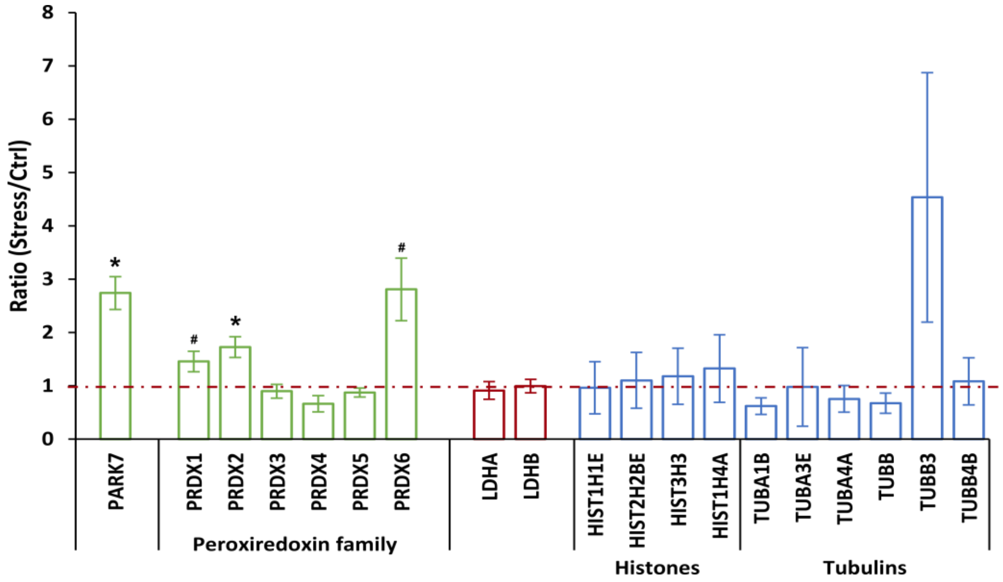


**Supplementary Figure 1 – Evaluation of the secreted levels of some groups of proteins associated with oxidative stress and potential markers of membrane disruption.** The protein evaluated were the protein DJ-1(PARK7), a redox sensor known to be secreted in higher levels during oxidative stress; the redo-related proteins from the peroxiredoxin family; two isoenzymes of the lactate dehydrogenase (LDHA and LDHB); and nuclear and structural proteins, namely histones and tubulins. Data correspond to the mean ratio ± SEM of four independent experiments. # and * indicate a $p < 0.1$ and $p < 0.05$, respectively, for statistically significant differences using the One-sample Student's t-test against a theoretical value of one.

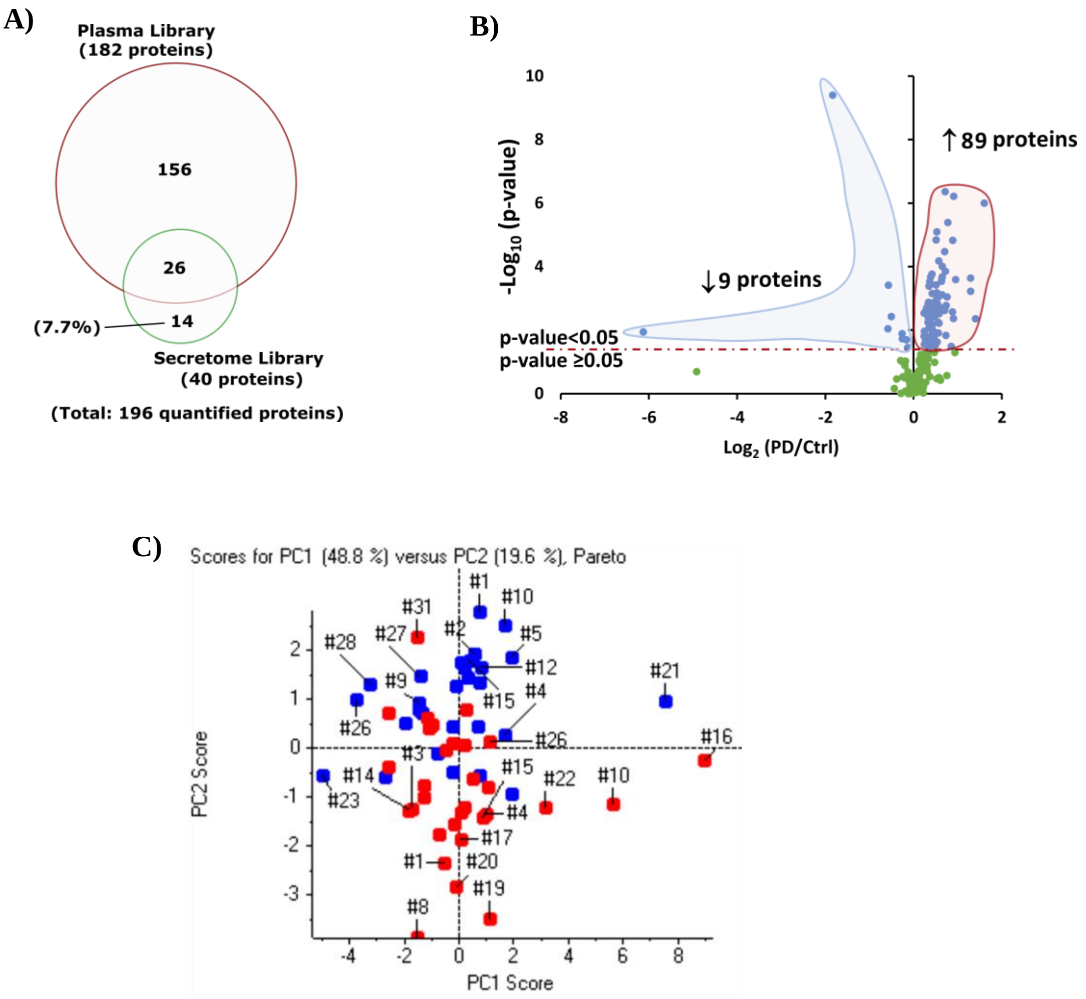


**Supplementary Figure 2 - Conventional untargeted proteome analysis of plasma samples of PD and Ctrl samples. A)** Venn diagrams comparing the library used in the extraction process considering all the proteins quantified. The combination of the two groups results in the quantification of 196 proteins in plasma, from those 14 were quantified in plasma only when considering the secretome library, which corresponds to an increase of 7.7%. **B)** Volcano plot representing the differential proteome analysis of the secretome between Ctrl and PD patients. From the 196 quantified proteins, a total of 9 proteins were significantly decreased in PD patients (blue shadow) and 89 proteins were significantly increased (red shadow). Statistical analysis was performed by Student's t-test and statistical significance was considered for p-values below 0.05 (blue dots). **C)** Principal component analysis using the replicate values of the 98 proteins significantly altered between the PD patients and controls (Figure 2H-I). The contribution of each component for explaining the total variance is indicated on top of the graphic. PCA processing was performed using the Pareto scaling, indicating a poor separation of the conditions (with the combination of the two components only explaining 68.4% of the separation).

**A)**

**Wilks' Lambda**

| Test of Function(s) | Wilks' Lambda | Chi-square | df | Sig. |
|---|---|---|---|---|
| 1 | .776 | 14.233 | 2 | .001 |

**B)**

**Classification Results** [a,c]

| | | Protein | Predicted Group Membership Ctrl | PD | Total |
|---|---|---|---|---|---|
| Original | Count | Ctrl | 22 | 6 | 28 |
| | | PD | 9 | 22 | 31 |
| | % | Ctrl | 78.6 | 21.4 | 100.0 |
| | | PD | 29.0 | 71.0 | 100.0 |
| Cross-validated [b] | Count | Ctrl | 21 | 7 | 28 |
| | | PD | 9 | 22 | 31 |
| | % | Ctrl | 75.0 | 25.0 | 100.0 |
| | | PD | 29.0 | 71.0 | 100.0 |

a. 74.6% of original grouped cases correctly classified.

b. Cross validation is done only for those cases in the analysis. In cross validation, each case is classified by the functions derived from all cases other than that case.

c. 72.9% of cross-validated grouped cases correctly classified.

**C)**

**Area Under the Curve**

Test Result Variable(s): Probabilities of Membership in Group 1 for ···

| Area | Std. Error [a] | Asymptotic Sig. [b] | Asymptotic 95% Confidence Interval Lower Bound | Upper Bound |
|---|---|---|---|---|
| .821 | .058 | .000 | .708 | .934 |

a. Under the nonparametric assumption

b. Null hypothesis: true area = 0.5

**Supplementary Figure 3 – SPSS output of the LDA and ROC curve analysis. A)** Test statistics for the discriminant model built from the combination of Clusterin and VPS35. **B)** Classification results including a cross-validation. **C)** Test statistics for the ROC curve determined for the LDA model created with the combination of the Clusterin and VPS35 proteins.

**A)**

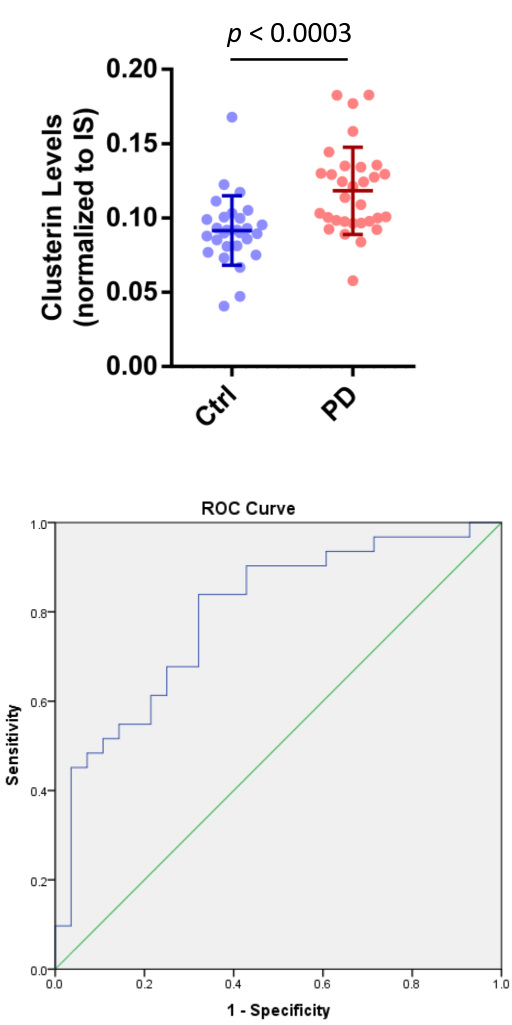


**Area Under the Curve**

Test Result Variable(s): CLUS_HUMAN

| Area | Std. Error[a] | Asymptotic Sig.[b] | Asymptotic 95% Confidence Interval | |
|---|---|---|---|---|
| | | | Lower Bound | Upper Bound |
| .795 | .059 | .000 | .680 | .910 |

a. Under the nonparametric assumption

b. Null hypothesis: true area = 0.5

**B)**

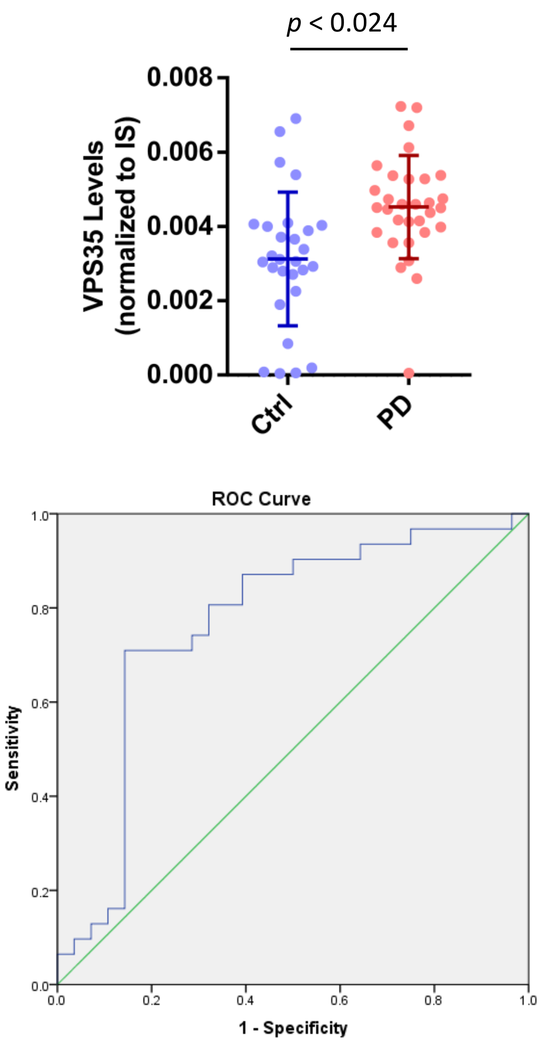


**Area Under the Curve**

Test Result Variable(s): VPS35_HUMAN

| Area | Std. Error[a] | Asymptotic Sig.[b] | Asymptotic 95% Confidence Interval | |
|---|---|---|---|---|
| | | | Lower Bound | Upper Bound |
| .767 | .066 | .000 | .637 | .897 |

a. Under the nonparametric assumption

b. Null hypothesis: true area = 0.5

**Supplementary Figure 4 – ROC curve analysis of each individual protein. A)** Distribution of the normalized Clusterin levels among the individuals tested (upper panel) and analysis of its individual diagnostic potential (lower panel). **B)** Distribution of the normalized VPS35 levels among the individuals tested (upper panel) and analysis of its individual diagnostic potential (lower panel). Plots of the ROC curves are indicated on top, and the respective test statistic indicated on bottom.

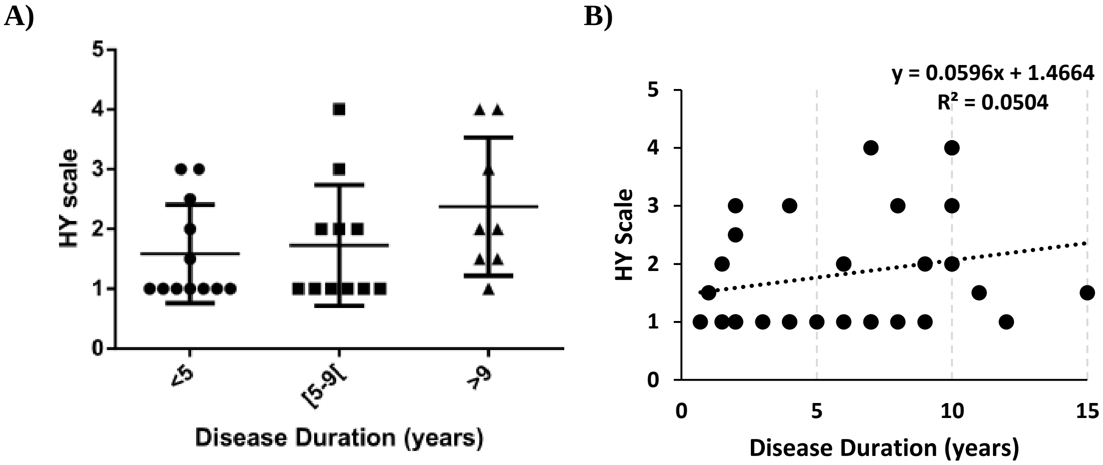


**Supplementary Figure 5 - Evaluation of the correlation between disease duration and HY scale of the PD group used in this work. A)** Distribution of the patients' HY scores according to the disease duration. Patients were grouped into 3 classes (until 5 years, 5 to 9 years and more than 9 years of disease duration). **B)** Scatter-plot of the HY scores considering the disease duration, with the respective trendline (dot-line), equation and coefficient of determination (R2). HY scores do not present a significant correlation with the disease duration (Spearman's Rank correlation coefficient (r) of 0.262 and a p-value of 0.154).

**A)**

| | Technical Replicates | Abs 1 | Abs 2 | Abs 3 | Av. | SD | %CV |
|---|---|---|---|---|---|---|---|
| **GFP** | Pool 1 | 0.161 | 0.177 | 0.236 | 0.191 | 0.040 | 20.6 |
| | Pool 2 | 0.153 | 0.151 | 0.153 | 0.152 | 0.001 | 0.8 |
| **Clusterin** | Pool 1 | 0.865 | 0.833 | 0.882 | 0.860 | 0.025 | 2.89 |
| | Pool 2 | 0.886 | 0.856 | 0.894 | 0.879 | 0.020 | 2.28 |
| **VPS35** | Pool 1 | 0.457 | 0.472 | 0.446 | 0.46 | 0.013 | 2.8 |
| | Pool 2 | 0.466 | 0.454 | 0.437 | 0.45 | 0.015 | 3.2 |

**B)**

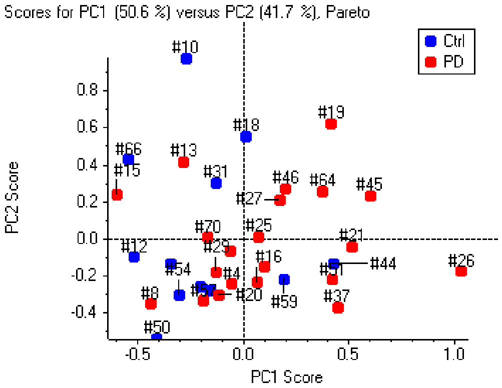


**Supplementary Figure 6 - ELISA results using an independent cohort of samples. A)** Evaluation of the variability of the quantification of the spiked IS, Clusterin, and VPS35 proteins by commercially available ELISA kits. The reproducibility of the measurements was evaluated by the analysis of the coefficient of variation (CV) of 3 technical replicates of four pooled samples (Pool 1 to 4). The median CV was 3%. **B)** Principal component analysis using the acquired absorbance values of IS, Clusterin and VPS35. The contribution of each principal component for explaining the total variance is indicated on top of the graphic. PCA processing was performed using the Pareto scaling.
