# Supplementary material for: A different vision of translational research in biomarker discovery: a pilot study on circulatory mitochondrial proteins as Parkinson’s disease potential biomarkers
Source: Transl Neurodegener. 2020 Apr 3;9:11. doi: 10.1186/s40035-020-00188-0 (PMC7118951; doi:10.1186/s40035-020-00188-0)
Supplement: Supplementary file 3 — Additional file 3: Supplementary Table 1. Data of patients and control individuals included in the discovery study. [file 40035_2020_188_MOESM3_ESM.pdf]

**Supplementary Table 1 – Data of patients and control individuals included in the discovery study.** Individual sample codes (in italic) and demographic parameters are indicated for all the samples. Clinical parameters are indicated in the disease group. For each group it is indicated an overall characterization of the group with the indication of the mean values, intervals, and gender distribution (indicated in bold).

| | | **Age at blood coll. (y)[a]** | **Gender[b]** | **Disease Duration (y)** | **Age at Onset (y)** | **Family history[c]** | **HY scale[d]** | **Total L-Dopa (converted CR + IR) (mg/day)[e]** |
|---|---|---|---|---|---|---|---|---|
| **Parkinson's Disease Group** | PD10 | 63 | F | 15.0 | 48 | N | 1.5 | 400 |
| | PD24 | 63 | F | 9.0 | 54 | Y | 2 | 475 |
| | PD35 | 63 | F | 1.5 | 62 | N | 2 | 300 |
| | PD11 | 64 | M | 2.0 | 62 | N | 1 | 300 |
| | PD38 | 64 | M | 10.0 | 54 | N | 2 | 400 |
| | PD32 | 67 | M | 10.0 | 57 | N | 4 | 300 |
| | PD34 | 68 | M | 5.0 | 63 | N | 1 | 200 |
| | PD39 | 69 | M | 1.5 | 68 | N | 1 | 300 |
| | PD33 | 70 | M | 2.0 | 68 | N | 1 | 0 |
| | PD23 | 71 | M | 10.0 | 61 | N | 2 | 300 |
| | PD29 | 72 | F | 1.0 | 71 | N | 1.5 | 150 |
| | PD30 | 72 | M | 6.0 | 66 | N | 1 | 400 |
| | PD14 | 73 | F | 4.0 | 69 | N | 1 | 300 |
| | PD16 | 73 | M | 7.0 | 66 | N | 1 | 200 |
| | PD15 | 74 | F | 7.0 | 67 | N | 1 | 300 |
| | PD18 | 75 | M | 0.7 | 74 | N | 1 | 50 |
| | PD8 | 76 | F | 2.0 | 74 | N | 3 | 733 |
| | PD17 | 76 | F | 8.0 | 68 | N | 1 | 850 |
| | PD7 | 77 | M | 10.0 | 67 | N | 4 | 1033 |
| | PD28 | 77 | M | 8.0 | 69 | N | 3 | 200 |
| | PD31 | 77 | M | 6.0 | 71 | N | 2 | 150 |
| | PD36 | 77 | M | 2.0 | 75 | N | 2.5 | 1150 |
| | PD6 | 78 | M | 3.0 | 75 | N | 1 | 650 |
| | PD19 | 79 | M | 4.0 | 75 | N | 1 | 650 |
| | PD21 | 79 | M | 12.0 | 67 | N | 1 | 0 |
| | PD26 | 80 | F | 7.0 | 73 | N | 4 | 300 |
| | PD27 | 80 | F | 4.0 | 76 | N | 3 | 200 |
| | PD12 | 81 | F | 11.0 | 70 | N | 1.5 | 725 |

| | | | | | | | | |
|---|---|---|---|---|---|---|---|---|
| | PD22 | 84 | F | 9.0 | 75 | N | 1 | 400 |
| | PD25 | 85 | F | 10.0 | 75 | N | 3 | 200 |
| | PD20 | 86 | F | 6.0 | 80 | N | 2 | - |
| | **Group overview** | **74±7 [63, 83]** | **M:17 (54.8%) F: 14 (45.2%)** | **6.2±3.8 [0.7, 15]** | **67.7±7.4 [48, 80]** | **N: 30 Y:1** | **1.8±1 [1, 4]** | **374.7±288.7 [0, 1150]** |
| **Control Group** | Ctrl29 | 55 | M | - | - | - | - | - |
| | Ctrl34 | 56 | F | - | - | - | - | - |
| | Ctrl12 | 57 | M | - | - | - | - | - |
| | Ctrl13 | 57 | F | - | - | - | - | - |
| | Ctrl14 | 57 | M | - | - | - | - | - |
| | Ctrl16 | 57 | M | - | - | - | - | - |
| | Ctrl17 | 57 | M | - | - | - | - | - |
| | Ctrl31 | 57 | F | - | - | - | - | - |
| | Ctrl32 | 57 | M | - | - | - | - | - |
| | Ctrl10 | 60 | M | - | - | - | - | - |
| | Ctrl27 | 60 | F | - | - | - | - | - |
| | Ctrl18 | 61 | M | - | - | - | - | - |
| | Ctrl24 | 62 | F | - | - | - | - | - |
| | Ctrl38 | 62 | F | - | - | - | - | - |
| | Ctrl15 | 64 | M | - | - | - | - | - |
| | Ctrl6 | 65 | F | - | - | - | - | - |
| | Ctrl7 | 70 | M | - | - | - | - | - |
| | Ctrl39 | 73 | F | - | - | - | - | - |
| | Ctrl26 | 74 | F | - | - | - | - | - |
| | Ctrl37 | 75 | F | - | - | - | - | - |
| | Ctrl28 | 77 | M | - | - | - | - | - |
| | Ctrl30 | 77 | M | - | - | - | - | - |
| | Ctrl35 | 78 | M | - | - | - | - | - |
| | Ctrl23 | 79 | M | - | - | - | - | - |
| | Ctrl11 | 81 | M | - | - | - | - | - |
| | Ctrl20 | 81 | F | - | - | - | - | - |
| | Ctrl36 | 81 | F | - | - | - | - | - |
| | Ctrl8 | 83 | M | - | - | - | - | - |
| | **Group overview** | **67±10 [55, 83]** | **M: 16 (57.1%) F: 12 (42.9%)** | - | - | - | - | - |

[a]Age at blood coll. (y): age at blood collection in years; [b]The individuals were divided into males (M) and females (F); [c]Number of individuals with familiar history of PD (Y) and those without any familiar link to the disease (N); [d]Hoehn and Yahr scale; [e]Total L-Dopa (converted CR + IR)

(mg/day): calculated as the controlled release (CR) plus immediate release (IR) in milligrams per day.
