# Supplementary material for: A different vision of translational research in biomarker discovery: a pilot study on circulatory mitochondrial proteins as Parkinson’s disease potential biomarkers
Source: Transl Neurodegener. 2020 Apr 3;9:11. doi: 10.1186/s40035-020-00188-0 (PMC7118951; doi:10.1186/s40035-020-00188-0)
Supplement: Supplementary file 4 — Additional file 4: Supplementary Table 2. Data of patients and control individuals used in the preliminary validation. [file 40035_2020_188_MOESM4_ESM.pdf]

Supplementary Table 2 – Data of patients and control individuals used in the preliminary validation. Individual sample codes (in italic) and demographic parameters are indicated for all the samples. For each group it is indicated an overall characterization of the group with the indication of the mean values, intervals, and gender distribution (indicated in bold).

		Age (years)	Gender
**Control Group**	*Ctrl10*	53	M
	Ctrl31	58	F
	Ctrl44	60	M
	Ctrl66	64	F
	Ctrl50	67	F
	Ctrl12	71	F
	Ctrl38	71	F
	Ctrl54	72	M
	Ctrl59	72	F
	Ctrl18	73	F
	Ctrl58	74	F
	Ctrl39	75	F
	**Group overview**	**67.5±7.2 [53, 75]**	**M: 3 (25%) F: 9 (75%)**
**Parkinson's Disease Group**	*PD3*	38	F
	PD51	46	M
	PD64	47	F
	PD16	49	F
	PD21	50	F
	PD8	50	F
	PD20	56	M
	PD27	57	M
	PD19	58	F
	PD25	58	M
	PD46	58	F
	PD70	60	F
	PD37	61	M
	PD4	63	F
	PD26	67	M
	PD57	68	M
	PD13	69	M

PD29	69	M
PD42	69	F
PD34	75	F
PD15	76	F
PD45	79	F
**Group overview**	**60.1±10.7 [38, 79]**	**M: 9 (40.9%) F: 13 (59.1%)**

[a]Age at blood coll. (y): age at blood collection in years; [b]The individuals were divided into males (M) and females (F).
